# The patient and clinician perspective on ‘early’ bowel resection for terminal ileal Crohn's disease (EBRIC): protocol for a multicentre mixed‐methods study

**DOI:** 10.1111/codi.70042

**Published:** 2025-03-02

**Authors:** Nilofer Husnoo, Lynda Wyld, Alan J. Lobo, Jenna L. Morgan, Deborah Hawkins, Louise Hunt, Nyantara Wickramasekera, Laura Marshall, Steven R. Brown

**Affiliations:** ^1^ University of Sheffield Sheffield UK; ^2^ Sheffield Teaching Hospitals NHS Foundation Trust Sheffield UK; ^3^ Doncaster and Bassetlaw Teaching Hospitals NHS Foundation Trust Doncaster UK; ^4^ Patient Representative Peterborough UK; ^5^ Sheffield Centre for Health and Related Research Sheffield UK

**Keywords:** biological therapy, Crohn's disease, ileocaecal resection, mixed‐methods, shared decision‐making

## Abstract

**Aim:**

Emerging evidence supports the consideration of surgery earlier in the treatment pathway for isolated luminal terminal ileal (TI) Crohn's disease (CD), as an alternative to medical therapy. Surgery is still considered late in the treatment pathway; recruiting participants into trials comparing medical therapy and surgery is difficult. This will be the first study to explore patients' and clinicians' views on bowel resection as an alternative to medical therapy for surgery‐naïve luminal TI CD. An understanding of the facilitators and barriers to this approach will provide insight into the gap between the evidence base and practice; these should be considered when designing future trials.

**Methods:**

A multicentre mixed‐methods study (NCT06116604) will be conducted. This will include semi‐structured interviews with 25–35 patients with TI CD exploring their views of treatment options, a survey of patients who have undergone a bowel resection for TI CD measuring their decision‐regret relating to the timing of their first resection (*n* = 271), and discrete choice experiments with healthcare professionals treating inflammatory bowel disease (surgeons, nurses and gastroenterologists) and with patients with TI CD (*n* = 100–300 for each participant group) to understand the importance given to different factors when making treatment choices. Patients will be recruited from 10 English and Welsh hospitals and healthcare professionals will be recruited from across the UK.

**Ethics and dissemination:**

This study has been approved by the London—Brent NHS Research Ethics Committee (reference 23/PR/0568). Dissemination will be through international and national colorectal and gastroenterology meetings and through the study patient panel.

## BACKGROUND

Crohn's disease (CD) has a prevalence of 1 in 650 in the UK [1]. The terminal ileum (TI) or ileocaecum is the most common location of CD, with a third of patients having isolated disease of the TI [2, 3]. Typically, CD is treated with medical therapy first, often with steroids to induce remission, followed by maintenance of remission with immunosuppressants. A bowel resection has long been seen as an option when medical therapy fails or to treat disease complications [4].

The optimal use of biologics (such as early intervention and ‘treat‐to‐target’ strategies) has been shown to improve outcomes [5, 6]. A decrease in the rate of surgical resections for CD has been reported since the introduction of biologics in clinical practice [7]. However, this has not been consistent [8, 9]; data from population‐based cohort and referral centre studies suggest that the decrease has only been modest [10]. Even in the modern biological era, up to 80% of patients with TI CD will require surgery eventually [11]. This is the basis for the argument that effective medical therapy perhaps delays surgery without preventing it all together. Equally, prolonged medical therapy without the desired effect can allow disease progression, leading to fibrotic stenoses or perforations, abscesses and fistulas [12]. The eventual operation is likely to be more technically challenging, with a higher risk of complications [13].

There is increasing evidence in favour of earlier surgery for uncomplicated luminal CD localised to the ileocaecum. Studies suggest that early bowel resection reduces the risk of disease relapse and leads to more durable remission compared with escalation of medical therapy [14, 15]. The LIR!C randomised controlled trial, comparing laparoscopic ileocaecal resection with infliximab for terminal ileitis in CD, has been a landmark study supporting the practice of surgery as an alternative to medical therapy [16]. At 1 year, surgery and medical treatment (infliximab) produced similar quality of life scores, although laparoscopic ileocaecal resection was more cost‐effective, a relevant point with the increasing economic pressures on healthcare delivery [17]. At 5 years, no patient in the surgery arm required a second resection and three‐quarters avoided further biologics, while nearly half in the infliximab arm underwent surgery [18]. Observational studies also indicate a reduced need for medical therapy and subsequent surgery in patients having early surgery compared to those on conventional medical therapy [19, 20, 21, 22, 23, 24].

Surgery for inflammatory bowel disease (IBD) has also evolved significantly over the past two decades, with an increasing number of dedicated IBD units, surgeons specialising in IBD and the increasing adoption of minimally invasive techniques and enhanced recovery protocols, all of which have led to significantly reduced morbidity rates [25].

## HYPOTHESIS, AIMS AND OBJECTIVES

There is sufficient evidence to support the consideration of early surgery as an alternative to medical therapy for isolated TI disease. However, it is acknowledged that the adoption of earlier surgery may be a challenge [26]. Patients often see surgery as a last resort, their fears of surgery being driven by the risk of a stoma and of surgical complications [27]. Recruitment into trials comparing early bowel resection with medical therapy for ileocaecal CD is also challenging [28, 29]. Guidelines vary in their precise recommendations on the positioning of surgery as an alternative to medical therapy [30, 31, 32]. This may reflect the lack of consensus, amongst clinicians and researchers, on how ‘early’ an operation should be offered to achieve optimal outcomes, and clinical practice is likely to vary.

We consequently hypothesise that, despite the evidence, surgery is still considered late in the treatment of isolated CD of the TI. Patients and their healthcare professionals (HCPs) are the two main stakeholders in the decision‐making process around treatment. The aims of this mixed‐methods study are therefore to determine

- patients' and clinicians' views on bowel resection as an alternative to medical therapy in the context of surgery‐naïve isolated TI CD, and
- the facilitators and barriers to ‘early’ surgery from a clinician and patient perspective.

The objectives are

- to understand the current positioning of surgery in the management of surgery‐naïve TI CD and the factors that influence the clinician's choice of therapy;
- to explore the patient perspective on the role and acceptability of medical therapy and surgery for TI CD and the factors that influence their treatment preferences;
- to explore patients' views on the timing of their first ileocaecal resection for CD and assess their decision‐regret relating to this choice;
- to elicit clinician and patient preferences for escalation of medical treatment versus surgery and their willingness to trade between them depending on the risks involved and outcomes of each option.

## METHODS

This protocol has been prepared according to the SPIRIT checklist [33] and is registered on the clinicaltrials.gov database (NCT06116604). The study is funded by grants from the Sheffield Hospitals Charity and Crohn's and Colitis UK. A patient panel was set up with funding support from the Research Design Service of the National Institute for Health and Care Research.

Little is known about patients' and HCPs' treatment preferences for TI CD and the potential barriers to earlier surgery. A mixed‐methods approach is therefore appropriate. Qualitative methods will allow in‐depth exploration of participants' views, generating novel insights. These can then be studied quantitatively using a larger sample size [34].

**FIGURE 1:**
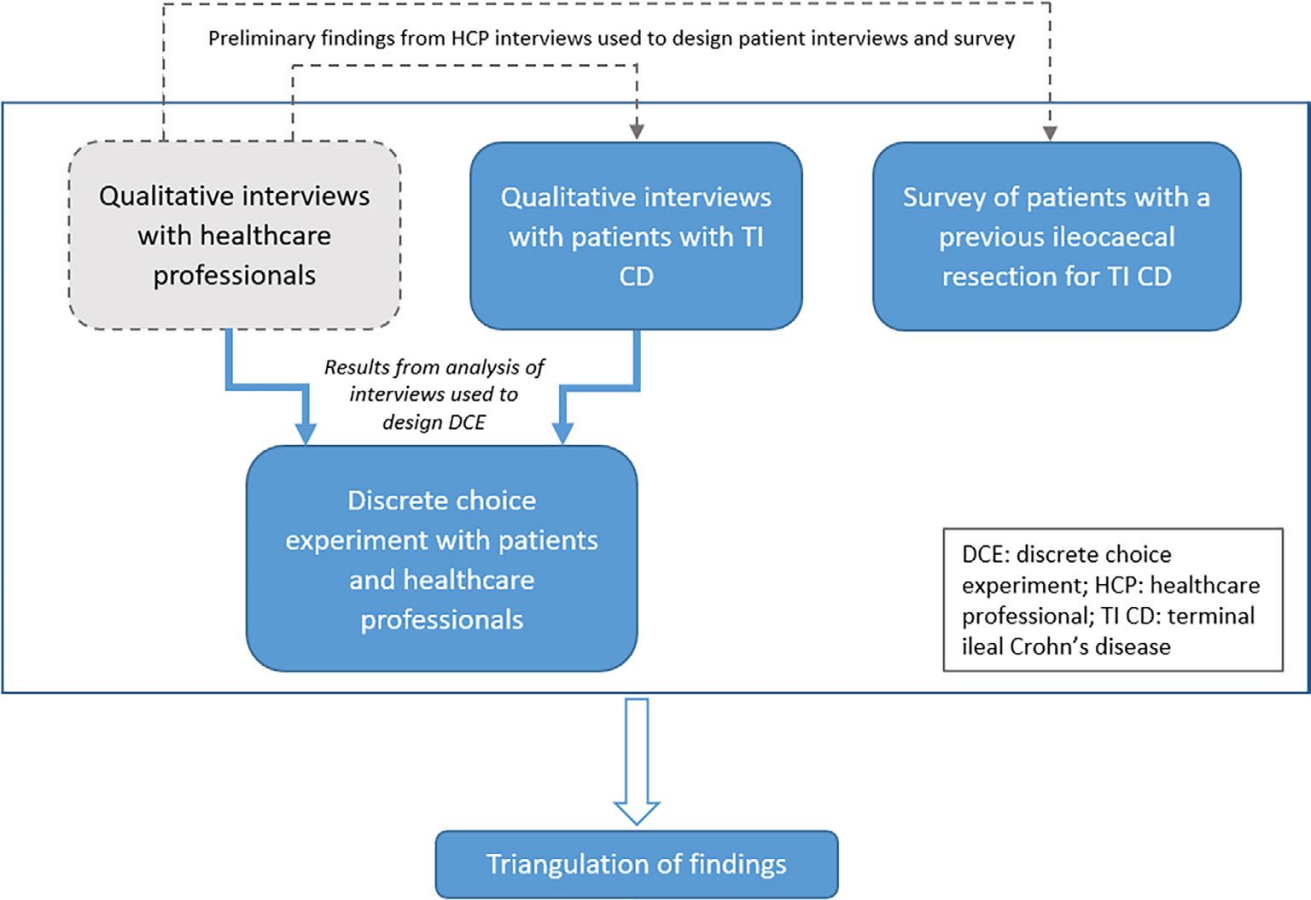
Schematic of the EBRIC study.

**FIGURE 2:**
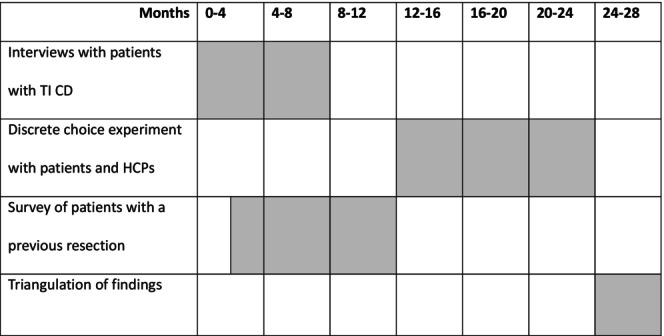
Proposed study timeline.

We have previously conducted semi‐structured interviews with HCPs with an interest in IBD as an exploratory study, the preliminary findings of which contributed to the design of the study described in this protocol. Figure 1 shows the overall schematic of the study. Figure 2 provides a proposed timeline for the conduct of the study.

### Participating centres

Eligible HCPs from across the UK will be recruited (for the discrete choice experiment (DCE)). For the patient components of the study, participants will be recruited from 10 National Health Service (NHS) hospitals across the UK to include five tertiary referral centres for IBD and five secondary care centres.

### Semi‐structured qualitative interviews with healthcare professionals

As described above, we have already conducted these interviews (data unpublished as of yet), the preliminary findings of which were used to design the rest of the study (described in sections up to Study management). A brief description of the methods used to conduct the HCP interviews has been provided below for context.

The aim of these interviews was to investigate the clinician perspective on the role of surgery for localised luminal ileocaecal CD, by qualitatively exploring how they make treatment‐related decisions and their views on ‘early’ surgery. The eligibility criteria were as described in Table 1.

**TABLE 1:** Eligibility criteria for the healthcare professional interviews.

Healthcare professional interviews: inclusion criteria
1. Gastroenterologist, or 2. Colorectal surgeon, or 3. Clinical nurse specialist with an interest or expertise in Ibd and working in an NHS centre in any part of the UK

Abbreviations: IBD, inflammatory bowel disease; NHS, National Health Service.

A purposive sample was recruited, based on job role (medical, surgical, nursing), level of specialist IBD care provided at the participant's unit (tertiary referral centre vs. secondary care) and geographical location to ensure a mix of experience and representation across the UK. Purposive sampling is a commonly used approach in qualitative research; participants are selected based on characteristics that allow detailed exploration of themes relevant to the research questions [35, 36]. Participants were recruited through social media networks and through the study team's contacts using a snowballing strategy (whereby participants helped with recruitment by identifying and contacting eligible candidates from their personal and professional networks [37]) and were contacted via email; electronic written consent was sought. Recruitment continued until data saturation was achieved (i.e., no new codes or information were identified in the data, which determines when further sampling and data collection should stop [38]). The interview schedule (File S1) was developed with input from patient and clinician experts and evolved iteratively throughout the conduct of the study. Interviews were conducted by NH (clinician, clinical researcher and PhD student), supervised by JLM, following training by JLM and LW (clinicians and experts in qualitative interview‐based research). Data were collected and analysed as they will be for the patient interviews (described in Semi‐structured qualitative interviews with patients below).

### Semi‐structured qualitative interviews with patients

Semi‐structured interviews will allow insight into patients' views on treatment for ileocaecal CD, on the acceptability of surgery as an alternative to medical therapy and into the factors influencing decision‐making.

#### Eligibility and recruitment

The eligibility criteria are outlined in Table 2. Restricting the study period to 10 years allows the inclusion of patients who had access to relatively similar and to current treatment options as management of CD continues to evolve. Patients will be identified by the Principal Investigator for each site from medical records or in inpatient and outpatient settings. Written consent (paper consent returned postally or electronic consent returned via email) will be obtained by a member of the local research team (i.e., not a clinician directly involved in their care to avoid coercion). Sampling will be purposive, based on whether participants are treated at a tertiary or secondary care centre, the type of treatment (medical vs. surgical) and, where possible, ethnicity and deprivation index of their postcode area to ensure representation from diverse socioeconomic and ethnic backgrounds. Previous work in the field shows that data saturation can usually be achieved with 15 interviews [39]. As both patients with experience of medical therapy only and patients with experience of surgery +/− medical therapy will be included, we anticipate needing to interview 25–35 patients but will stop recruitment when data saturation is achieved.

**TABLE 2:** Eligibility criteria for the patient interviews.

Patient interviews: eligibility criteria
*Inclusion criteria* 1. ≥18 years of age, with current or previous TI or ileocaecal CD (with or without involvement of the right colon). Where the patient also has concomitant CD affecting other sites, this should be TI‐dominant disease (i.e., treatment mainly required for TI disease); AND 2. The diagnosis of TI CD should have been made between 6 months and 10 years prior to the launch of the study; AND 3. Experience of steroid‐sparing treatment for TI CD to include enteral nutrition, immunomodulator therapy or biologics, or bowel resection (ileocolic resection or right hemicolectomy) for TI CD (with or without a stoma)
*Exclusion criteria* 1. No previous treatment for TI CD or experience of steroids only as treatment 2. Unable to communicate in English (due to lack of resources to enable translation) 3. Lacking the mental capacity to take part in the study (e.g., memory impairment, dementia)

Abbreviations: CD, Crohn's disease; TI, terminal ileal.

#### Data collection

The interview schedule (File S1) is based on evidence from the literature and preliminary findings from the HCP interviews, with input from clinician and patient experts. Interviews will be semi‐structured and conducted via videoconference or telephone by researchers at the central site (NH, alongside a medical student trained in the conduct of qualitative interviews). Each will last up to an hour and be digitally recorded.

#### Data analysis

All interviews will be transcribed verbatim by an external company specialising in medical transcription services (https://www.sterlingtranscription.co.uk/). Pseudo‐anonymised linked data will be entered into the latest version of NVivo (Pro) software (qualitative data analysis software). They will be coded by the researchers. At least 10% of the interviews will be coded independently by two researchers to ensure intercoder reliability. Data will be thematically analysed using a primarily inductive approach as outlined by Braun and Clarke [40, 41]. Themes and subthemes will be generated, and data will be organised in a matrix as described by the framework method for ease of interpretation [42].

Data analysis will start after the first five interviews have been completed to allow the identification of new themes. The interview schedule will evolve iteratively, before further data collection and analysis takes place involving a few more participants. This process will continue until data saturation is achieved.

### Discrete choice experiment (DCE)

DCEs are increasingly used to investigate preferences in healthcare [43]. They present the respondent with hypothetical choice sets consisting of at least two competing alternatives (e.g., two treatment options). The choice sets vary along selected attributes (e.g., characteristics of each treatment option, or risk of an outcome with each treatment); in each set, the respondent is asked to choose their preferred alternative [43]. The aim of this DCE will be to ascertain the relative weights HCPs and patients attach to selected variables involved in the decision‐making process and their willingness to trade between them when choosing between medical therapy and surgery for TI CD. This will provide insight into the treatment‐related factors and outcomes that are valued over others, whether preferences vary between patients and HCPs, and why one treatment option might be preferred over another.

#### Design

Potential key attributes (such as chance of drug requirement, stoma formation and disease recurrence) that are important to both HCPs and patients will be identified from the analysis of the interviews. Qualitative research methods are commonly used to select DCE attributes [44]. Discussions with the Study Management Group (SMG) and patient panel will confirm the selection of attributes to be included in the DCE from this list of potential attributes. Levels (e.g., risk reduction associated with each treatment) will be confirmed using data from the literature. Should the preferences of the HCPs and patients overlap significantly, a single choice experiment will be conducted with both, using appropriate language for each group; otherwise two separate choice experiments will be designed.

Ngene software will be used to generate a D‐efficient design, following the principles of minimum overlap, orthogonality and level balance [45]. Around 8–12 choice sets will be included in the survey to avoid cognitive burden. The HCP choice experiment questionnaire will be piloted with three to four members of the colorectal, gastroenterology and IBD specialist nursing team at the host institution. The patient questionnaire will be piloted with a focus group of three to four patients at the host institution. Appropriate modifications will be made based on feedback.

#### Sampling

Clear guidelines are lacking on methods to calculate sample sizes, which vary substantially from 100 to 600 [57, 58]. At this stage, the sample size cannot be calculated without knowledge of the attributes and levels. We will adopt the formula used by Orme to estimate the sample size necessary to achieve a tolerable margin of error [46]. A number greater than 100 is usually recommended as it ensures a basis for modelling preference data [47]. Having close to 300 responses may allow for subgroup analyses, based on our team's experience of conducting DCEs. A minimum of 150 responses and up to 300 responses per DCE will be aimed for.

Questionnaires will therefore be distributed to 385–770 patients across the 10 participating sites, assuming a response rate of 39% [59]. For the HCP component, we will use recruitment methods described below to obtain at least the minimum number of responses required for meaningful statistical analyses. Final sample size calculations will be performed once data from both patient and clinician interviews have been fully analysed to identify relevant attributes and levels.

#### Eligibility and recruitment

Eligibility criteria will be the same as for the qualitative interviews (Table 1). Eligible HCPs will be recruited by the central study team through a combination of professional societies, social media and the study team's personal contacts (using a snowball sampling strategy). HCPs will be emailed a link to an online information sheet and questionnaire. Reminder emails will be sent at 3–4 week intervals (with up to three reminders per participant). When promoting on social media, a direct link to the questionnaire will be provided. The anonymous nature of responses will be emphasised in the information sheet and the number of reminders sent will be restricted as described to minimise the risk of coercion.

Patients will be approached in inpatient and outpatient settings and given the questionnaire, with the integrated information sheet and consent form. Patients will also be identified from medical databases and will be sent the questionnaire by post or will be emailed a link to the online information sheet and questionnaire. Each patient will be given a unique participant number to enter on their questionnaire (pseudo‐anonymised linked) to allow the research teams to track non‐responders. Postal or email reminders will be sent at 4‐week intervals to non‐responders (up to two reminders), with a telephone call prior to the second reminder.

#### Data collection

The questionnaire will contain 8–12 hypothetical choice tasks and will ask patients and clinicians to make choices between two sets of treatments, for example biologic agents versus surgery, with varying levels of attributes provided such as ongoing need for medication, likelihood of subsequent surgery and risk of a stoma.

For HCPs, demographic information about participants and their centre will be collected. Participants will be asked to estimate the number of ileocaecal resections for CD undertaken in a year in their unit to gauge whether theirs is a high or low volume centre for IBD surgery, using arbitrary definitions previously adopted in a national audit [48]. Similarly for patients, data on demographics, duration of disease, current and previous treatments, current disease control via the IBD‐Control‐8 questionnaire [60], and health related quality of life via the EQ‐5D‐5L [49] will be gathered. These data will allow us to model the differences in preferences based on particular participant characteristics.

The online questionnaire will be hosted on Qualtrics, which is a secure online platform enabling participants to complete the questionnaire on a computer or mobile device.

#### Data analysis

Patient and clinician DCEs will be analysed separately. Responses will be modelled using a conditional logit model, commonly used for the analysis of choice data using the latest version of Stata [50]. Regression coefficients will be used to estimate the relative importance of attributes; marginal rates of substitution will be calculated (i.e., trade‐off preferences for treatments). Latent class models will be used to analyse individual heterogeneity and to identify subsets of participants with varying preferences if sample sizes allow [51]. Subgroup analyses according to whether patients have had previous surgery will identify the effect of previous therapy on their preferences for outcomes.

### Survey of patients who have undergone a previous bowel resection

The survey will explore patients' views on the timing of their first ileocaecal resection for CD and assess their decision‐regret and how this relates to preoperative and postoperative experiences. A similar study in 1994 showed that 75% of respondents would have preferred to have surgery a median of 12 months earlier than they actually did [52]. This study has not been repeated in the biologics era and patients' decision‐regret relating to ileocaecal resection for CD has never been assessed.

The primary outcomes are

1. patient preference for timing of resection and
2. decision‐regret (using a previously validated scale [53]).

The secondary outcome is correlation of the decision‐regret score with

1. selected preoperative treatment experiences (including duration of disease and experience of biologics);
2. shared decision‐making (assessed using the CollaboRATE tool [61]);
3. body image score, using the modified Hopwood Body Image Scale validated for use in IBD patients [62, 63] coupled with a cosmetic scale previously used in studies evaluating body image in patients undergoing ileocolic resection for CD [16, 64];
4. selected postoperative outcomes, including formation of a stoma, complications, disease relapse.

#### Design

The questionnaire content is based on themes identified in the literature (including a systematic review of early surgery compared with ongoing medical therapy previously conducted by the study team [15]) and from the interim analysis of our interviews with HCPs as well as input from the study team and clinician and patient experts. The questionnaire will be piloted with three to four patients at the host institution, and appropriate subsequent modifications made.

#### Eligibility and recruitment

The eligibility criteria are outlined in Table 3. An initial time frame of 5 years was chosen for the study period to limit recall bias; this has been changed to 7 years as the number of procedures performed during the COVID‐19 pandemic may have been significantly lower than usual. Patients will be identified from electronic records in each participating centre using procedure codes, pathology records and medical notes. Questionnaires will be posted to eligible participants or a link to the online version (on Qualtrics) will be emailed. Questionnaires will also be handed out or emailed to eligible patients when identified by clinicians in inpatient and outpatient settings.

**TABLE 3:** Eligibility criteria for patient survey.

Inclusion criteria for patient survey	Exclusion criteria for patient survey
1. Any patient who had their first ileocaecal or ileocolic resection or right hemicolectomy for TI CD (confirmed using the pathology report for the resected specimen) within up to 7 years preceding the launch of the study. Patients who subsequently require a redo ileocolic resection (such as for disease recurrence) during the 7 year study time frame chosen can still be included as long as their first resection was within the study period; AND 2. age 18 years and above; AND 3. resection with primary anastomosis or with a stoma; AND 4. procedure performed on an elective, emergency or semi‐elective basis.	1. Ileocaecal or ileocolic resection or right hemicolectomy performed as an emergency operation for the first presentation of CD (i.e., in someone not previously diagnosed with CD) 2. Incidental diagnosis of CD as a result of resection performed for an alternative pathology 3. Resection in the 3 months preceding recruitment into the study 4. Unable to communicate in English (due to lack of resources to enable translation) 5. Lacking the mental capacity to take part in the study (e.g., memory impairment)

Abbreviations: CD, Crohn's disease; TI, terminal ileal.

#### Sampling

We are not aware of any study that has measured the decision‐regret following bowel resection in CD patients. The sample size has therefore been calculated based on the estimated target population size. This study will be carried out across the 10 participating centres. A tertiary centre performs around 20 ileocaecal resections for CD in a year, and a secondary care centre around 10–15 [54]. It is expected that 20% of these are repeat resections. This equates to 130 first‐time resections annually across the 10 participating sites (using average values). A random sample of 271 therefore enables estimation of proportions within a ±5% margin of error, with 95% confidence. Response rates to patient surveys can be as low as 39% [59]. Therefore, 695 surveys will be distributed: 53 per secondary care centre and 86 per tertiary centre. The target sample size may need to be adjusted during the course of the study when more accurate estimates of the population size are obtained.

#### Data collection

Data will be collected on demographics, duration of disease, details of the procedure, postoperative complications, stoma formation and subsequent medical therapy, as well as on primary and secondary outcomes. Each patient will be given a unique participant number to enter on their questionnaire (pseudo‐anonymised linked). Non‐respondents will be sent reminders at 4–6 week intervals (maximum of two reminders) as well as a telephone call prior to the second reminder.

#### Data analysis

Quantitative results from the survey will be presented using descriptive statistics. Correlation analysis will be performed to determine factors related to increasing or decreasing levels of decision‐regret. Analyses will be performed using the latest version of SPSS. Qualitative data from open‐ended questions will be entered into the latest version of Nvivo (Pro) and will be thematically analysed.

### Triangulation

An integrated set of findings will be generated at the end of the study. The triangulation protocol developed by Farmer and colleagues will be adapted and used as described below [55].

1. Findings from each dataset will be sorted to identify key themes.
2. The themes will then be ‘convergence coded’ to determine if there is ‘agreement’, ‘silence’ or ‘dissonance’ between the findings from each dataset.
3. A global assessment of the degree of convergence will be made.
4. The unique themes in each dataset will be compared to enhance the completeness of integrated findings.
5. A plan will be made on how disagreements between researchers will be handled so that final decisions on the interpretation of findings can be reached.
6. The findings from this process will be shared with the SMG and the patient and public involvement (PPI) panel for their review and feedback.

This process will be carried out with the SMG (see below), other members of the wider study team, and members of the PPI panel, ensuring representation from all relevant stakeholders (colorectal surgeons, gastroenterologists, IBD nurse specialists, patients and researchers with expertise in qualitative and mixed methodology).

## STUDY MANAGEMENT

### Data management

All data will be handled and stored in accordance with the Data Protection Act 2018 and the GDPR 2018 principles. Access to physical and electronic data will be limited to appropriate members of the research team only. Original paper consent forms and questionnaires will be stored in the investigator site file at each local site. The key to pseudo‐anonymised linked patient questionnaires will be recorded on the enrolment log in the investigator site file at each site and will only be accessible to the local research team. This will allow the identification of non‐respondents. Electronic data and consent forms will be stored and processed on a secure drive using password‐protected NHS computers at the sponsor organisation. Study data will be archived for 5 years after the end of the study.

### Roles and responsibilities

The SMG—also authors of this study—will comprise the Chief Investigator (NH) and co‐investigators (to include clinicians with expertise in IBD (AJL, LM, SRB) and researchers with expertise in qualitative and mixed methodology (LW, JLM, AJL, SRB) and DCEs (NW)), as well as a representative from the patient panel (LH). The SMG will meet on a regular basis to monitor aspects of the conduct and progress of the research, ensuring that the protocol is adhered to. A local Principal Investigator will be named at each site to oversee the conduct of the study locally; they will liaise regularly with the Chief Investigator and study coordinator at the sponsor site (coordinating centre).

### Ethics

Ethical approval to conduct the study has been provided by the London—Brent NHS Research Ethics Committee (ref. 23/PR/0568). Health Research Authority approval has also been provided. Protocol amendments will be approved by ethics and the Health Research Authority and communicated to all local research teams by the central study team. Capacity and capability will be confirmed at each site before the study is initiated locally.

### Patient and public involvement (PPI)

A patient panel comprising patients with CD of the TI was set up. They have contributed to the development of the protocol and study documents. They have reviewed participant‐facing documents (information sheets and questionnaires) and pilot‐tested patient questionnaires. One member of this group was a co‐applicant on the Crohn's and Colitis UK grant, is a member of the SMG and a co‐author of this paper. We will hold regular meetings with this group, timed to coincide with key stages of the project. They will also be involved in the promotion of the study on public forums including social media and in the dissemination of findings.

### Dissemination

Study findings will be presented at national and international meetings, published in peer‐reviewed journals and communicated on social media platforms. These will also be communicated to all local Principal Investigators. We will work collaboratively with our patient panel to develop dissemination materials that are accessible and meaningful to patients.

## DISCUSSION

Since the launch of our study, results from the PROFILE study have been published. This trial demonstrated that the very early introduction of biologics in the treatment of CD leads to a significantly better steroid‐free and surgery‐free remission than the more conventional accelerated step‐up regime at 1 year. These findings are likely to impact HCPs' views on the relative positioning of surgery and medical therapy. However, the longer‐term outcomes of this approach are still unknown. Equally, surgical techniques for the treatment of ileocaecal CD continue to improve; new anastomotic techniques and/or radical excision of the mesentery may potentially reduce disease recurrence rates after ileocaecal resection [65, 66]. Our study will provide useful insights into patients' treatment preferences and values which, in the face of rapidly evolving medical and surgical approaches (and the resulting lack of clarity on where the equipoise lies), are an important consideration in the shared decision‐making process about treatment.

Additionally, whether earlier or later surgery for ileocaecal CD leads to better outcomes remains a priority research question [56]. Although the LIR!C study provided evidence in favour of surgery as an alternative to escalation to infliximab, surgery as an alternative to medical therapy at other points in the treatment algorithm could also potentially lead to more favourable outcomes. However, the difficulties in conducting a randomised trial comparing these two treatment modalities means that significant progress is yet to be made on addressing this question. We are unaware of any study conducted to investigate the potential barriers to earlier surgery. The findings from our study will be the first to explore the acceptability of earlier surgery amongst patients and HCPs and will shed light on the motives behind patients' and HCPs' treatment preferences. In so doing, we will also be able to explore factors that need consideration when designing subsequent trials comparing surgery and medical therapy for TI CD, including HCPs' and patients' views on the existence of clinical equipoise in this context.

## AUTHOR CONTRIBUTIONS

**Nilofer Husnoo:** Conceptualization; funding acquisition; writing – original draft; methodology; writing – review and editing. **Lynda Wyld:** Conceptualization; funding acquisition; methodology; validation; writing – review and editing; supervision. **Alan J. Lobo:** Conceptualization; funding acquisition; methodology; validation; writing – review and editing; supervision. **Jenna L. Morgan:** Conceptualization; funding acquisition; methodology; validation; writing – review and editing; supervision. **Deborah Hawkins:** Validation; methodology; writing – review and editing; project administration. **Louise Hunt:** Funding acquisition; writing – review and editing; methodology. **Nyantara Wickramasekera:** Writing – review and editing; methodology; software. **Laura Marshall:** Methodology; writing – review and editing; resources. **Steven R. Brown:** Funding acquisition; conceptualization; writing – review and editing; methodology; validation; resources; supervision.

## FUNDING INFORMATION

The study is funded by grants from the Sheffield Hospitals Charity and Crohn's and Colitis UK awarded to NH.

## CONFLICT OF INTEREST STATEMENT

AJL receives speaker and/or consulting fees from Takeda, BMS, Abbvie, Medtronic, Vifor, Janssen, Sandoz, MSD, Pfizer and Shield Therapeutics. The study sponsor and funder have had no role in the study design and writing of the report.

## ETHICS STATEMENT

Ethical approval to conduct the study has been provided by the London—Brent REC (ref 23/PR/0568).

## Supporting information


Data S1.

## Data Availability

Access to full protocol and statistical codes can be provided upon reasonable request to the corresponding author.
