# Supplementary material for: The patient and clinician perspective on ‘early’ bowel resection for terminal ileal Crohn's disease (EBRIC): protocol for a multicentre mixed‐methods study
Source: Colorectal Dis. 2025 Mar 2;27(3):e70042. doi: 10.1111/codi.70042 (PMC11873672; doi:10.1111/codi.70042)
Supplement: Supplementary file 1 — Data S1. [file CODI-27-0-s001.docx]

## Original healthcare professionals interview schedule

**The questions relate to patients with isolated terminal ileal or ileocaecal Crohn’s disease (L1 phenotype) without prior surgery. This excludes patients with very clear indications for surgery, for example symptomatic fibrotic stricture, or with very clear indications for medical therapy, for example multiple diseased segments involving a significant length of bowel.**

1. **What is your general approach to managing a patient with a new diagnosis who does not respond fully to steroids or relapses quickly off steroids?**

Prompts: What factors would make you consider immune modulators?

What factors would make you consider biological therapy?

What would make you consider offering an ileocaecal resection?

1. **You have mentioned some of the factors that guide your management. What are the other factors that influence your choice of therapy in this scenario?**

Prompts: *Patient factors*

Age of patient

Co-morbidities

Smoking status

Patient choice

*Disease factors*

Duration of disease

Duration of previous treatments

Number of courses of steroids

High risk disease features (e.g. perianal disease, fistulating disease)

Risk of relapse or disease recurrence

Development of complications/disease behaviour

Extra-intestinal manifestations

*Organisational and other factors*

Guidelines, including local guidelines

Costs of treatment

IBD MDT meeting discussions

Availability of joint clinics

Research evidence

Own experience

For gastroenterologists and surgeons: Nurse specialist’s input

For gastroenterologists & IBD CNS: Ease of involving surgical colleagues and relationship with them

For surgeons: Gastroenterology colleagues’ threshold for involving surgeons in the management

1. **In your view, what are the risks and benefits of continuing medical therapy and those of performing a bowel resection in this patient at this stage in their treatment?**

Prompts: Side effects & safety profile of medical therapy

Quality of life

Duration of benefit

Why would you not offer surgery at this stage? (Complications of surgery, recurrence, acceptability to patient)

Is the likelihood of medically treated patients needing surgery in next 12 -24 months a consideration?

Is the likelihood of surgically treated patients needing antiTNF in next 12 -24 months a consideration?

1. **When should a bowel resection be offered to a patient in this cohort? Where does it fit in in the management algorithm?**

Prompts: Complications of disease

Frequent relapses, hospitalisations

Steroid dependent/ steroid refractory disease

Once immunosuppressants have been trialled for a given period of time

Failure of medical therapy

Unacceptable side effects

Patient’s choice

1. **When would you first discuss surgery with this patient, and how would you approach this discussion?**

Prompts: In what circumstances would an in-depth discussion of surgery be had and formally offered as a treatment option?

What criteria would lead you to refer a patient to the IBD joint clinic for consideration of surgery?

1. **In scenarios where you feel that both ongoing medical therapy and an ileocaecal resection are valid options, what role does the patient play in decision-making?**

Prompts How is that input obtained?

1. **How do you think patients feel about an ileocaecal resection for limited TI disease?**
2. **Any additional comments**

## Original E-BRIC Interview schedule for patient interviews

1. Tell me about yourself (your age and how long you have been diagnosed with Crohn’s)

**Prompts:** How did you first find out that you have Crohn’s disease?

What symptoms have you experienced and what problems has it caused you?

What treatment have you had so far?

Have you had an operation? What operations have you had so far?

1. Can you tell me how you found out about your diagnosis and treatment options?

**Prompts**: Who explained the diagnosis? What did they say?

When you were first diagnosed, what were you told about your treatment options?

What were you told about the medications that you may need to take? Can you recall what you might have been told likelihood of the medication working? Side effects? What might happen if they don’t work?

How did you mostly find out about treatment options? (Clinicians/ internet/ charity organisations/ friends and family/ support groups)

1. Choice - There are multiple treatment options for Crohn’s disease. Have you ever been presented with a choice where your clinician and you have had to decide which option is most suitable?

**Prompts:** What influenced your decision-making process?

Pre-existing knowledge of treatment/ disease severity/ symptoms/ mode of administration/ impact on personal or professional life/ clinician influence/ friends or family/ side effects/ expected outcome of treatment in terms of stability of remission or symptom control or future need for medication or need for surgery

What outcomes are important to you? What do you want your treatment to achieve? (Quality of life, being able to eat normally, return to work, not taking medication, avoiding a stoma, not having an operation, avoiding hospitalisations, symptom control – pain, diarrhoea, positive body image, feeling less fatigued)

What outcomes or risks are you less concerned about/ do you find less relevant?

1. Surgery as a treatment option

**Prompts**: For surgery-naïve patients: What do you know about surgery as a treatment option?

How did you find out about surgery as a treatment option? When was it discussed for the first time? Who discussed it for the first time? Can you recall what they said?

What was/is your understanding of the aims of an operation?

Can you recall what was said about risks and benefits?

How did you feel/do you feel about potentially needing an operation? What were/ are your concerns or worries or hopes and expectations?

For surgery-naïve patients: What would make you consider an operation?

For patients with a previous resection: What made you consider an operation?

For patients with a previous resection: having had an operation, how well would you say that the information that you were given prior to your operation prepared you in terms of what to expect? Was there anything else that you wish you knew?

For patients who have had a resection: can you describe your experience of managing your disease after surgery? How does this compare to before you had an operation?

How do you feel about post-operative care and monitoring? i.e. ongoing need for medication, surveillance with endoscopy, potential future surgery?

1. Views on early bowel resection

**Prompts:** For surgery-naïve patients – would you consider an operation, knowing that there are still drugs that you have not tried/ can still be tried to control your symptoms?

Reasons for wanting to exhaust all medical options/ for wanting an operation while medical options still exist

What would make you choose one option over the other?

For patients with a previous resection – benefits or drawbacks of an operation compared to medical therapy. Do you wish you had your operation sooner? Why/ why not?
